# Comparison of Peyton's Four-Step Approach and Rapid Cycle Deliberate Practice in Basic and Advanced Airway Management: A Simulation-Based Study

**DOI:** 10.7759/cureus.90113

**Published:** 2025-08-14

**Authors:** Killen H. Briones-Zamora, Killen H. Briones-Claudett, Pryscilla Díaz Mora, Enny Berrus Zhumi, Ana Polanco Montero, Wilman E. Serrano Torres, Jaime Benites Solis, Anahi D. Briones-Zamora, Diana C. Briones-Marquez

**Affiliations:** 1 Simulation Clinic (SIMUEES), Universidad de Especialidades Espíritu Santo (UEES), Samborondón, ECU; 2 Medical Education and Research, Briones PulmoCare, Guayaquil, ECU; 3 Intensive Care Unit, Omni Hospital, Guayaquil, ECU

**Keywords:** endotracheal intubation, fiberoptic bronchoscope-guided intubation, fiberoptic intubation, laryngeal mask airway, peyton’s four-step approach, rcdp, simulation-based training

## Abstract

Background

Simulation-based training plays a critical role in the acquisition of airway management skills in undergraduate medical education. Peyton’s Four-Step Approach (PFSA) and Rapid Cycle Deliberate Practice (RCDP) are two structured, evidence-based methodologies used in simulation training. While both have shown educational benefits, few studies have directly compared their effectiveness across both basic procedures, such as laryngeal mask airway (LMA) placement and endotracheal intubation (ETI), and complex procedures, such as fiberoptic intubation (FOI), especially in time-limited massed practice formats.

Methods

Fifty-nine third-year medical students with no prior experience in airway management participated in this simulation-based study. They were divided into two cohorts: one trained using RCDP and the other using PFSA. Each cohort received three weekly sessions focused on LMA, ETI, and FOI, respectively, with individualized hands-on training. Final evaluations were conducted using the Objective Structured Assessment of Technical Skills (OSATS) for LMA and ETI, a Global Rating Scale (GRS) for bronchoscope handling, and a 10-item checklist for FOI. Statistical analysis included t-tests, Mann-Whitney U, chi-squared or Fisher’s exact tests, and Cronbach’s alpha for instrument reliability.

Results

PFSA resulted in significantly higher OSATS scores for LMA placement (38.06 ± 6.67 vs. 31.93 ± 7.70, p = 0.0017), with a higher proportion of non-competent participants in the RCDP group (p = 0.009). In contrast, RCDP outperformed PFSA in ETI OSATS scores (67.00 ± 12.32 vs. 58.93 ± 11.87, p = 0.012) and in the proportion of students achieving mastery (p = 0.013). For FOI, RCDP led to significantly better results on the GRS (14.73 ± 4.65 vs. 9.66 ± 2.79, p < 0.0001) and the procedural checklist (median: 6.0, interquartile range (IQR): 5-7 vs. median: 4.0, IQR: 3-6, p = 0.0021). No participants reached mastery in FOI, but the RCDP group had significantly more moderately competent students (p = 0.0194) and fewer non-competent participants (p = 0.0003). Reliability of assessment instruments was confirmed with Cronbach’s alpha values above 0.70.

Conclusion

PFSA was more effective for teaching basic skills such as LMA placement, likely due to its stepwise instructional design. RCDP showed superior outcomes in ETI and FOI, supporting its use in complex procedures that benefit from iterative practice and immediate feedback. These findings highlight the importance of tailoring simulation-based training methods to the complexity of the procedure and the learners’ needs, particularly in programs with limited training time, and underscore the value of incorporating follow-up assessments to evaluate long-term retention and the durability of training effects.

## Introduction

Simulation-based training in airway management is integral to undergraduate medical education, fostering the integration of theoretical knowledge with technical skills, both essential for clinical competence. Recent research has demonstrated that simulation outperforms traditional didactic methods, producing superior cognitive acquisition, psychomotor performance, and learner satisfaction. Participants consistently exhibit significant improvements in both skill and confidence following simulation-based instruction [1,2].

Competency in airway procedures is essential for physicians to manage airway emergencies effectively. Endotracheal intubation (ETI) remains the gold standard for airway management, especially in critical or life-threatening situations. The laryngeal mask airway (LMA) serves as an alternative when ETI is challenging or when rapid airway control is needed [3,4]. Fiberoptic intubation (FOI) is also considered a highly effective method for managing difficult airways, especially in awake patients, with success rates of 98% or higher [5]. This technique is particularly useful when anatomical or physiological issues like birth defects, tumors in the head or neck, upper airway injuries, or problems with the cervical spine make regular laryngoscopy hard to perform [3,4,6]. This method allows accurate tube placement before anesthesia, avoiding breathing issues or failed intubation [6]. However, it is a complex procedure; many professionals lack confidence using it, and since it is rarely done, with only 1% of cases, there is no standard training, making it hard to maintain the skill over time [6,7].

Rapid Cycle Deliberate Practice (RCDP) and Peyton’s Four-Step Approach (PFSA) are structured, evidence-based approaches commonly used in simulation education. RCDP involves brief, intensive practice sessions with immediate, targeted feedback, enabling learners to correct errors in real time and achieve competence before advancing [8]. It often incorporates massed practice, facilitating concentrated repetition within single sessions to accelerate skill acquisition. Conversely, PFSA employs a sequential framework-demonstration, deconstruction, comprehension, and performance that scaffolds procedural learning, particularly in small-group or individualized contexts [9].

However, few studies have directly compared the instructional effectiveness of RCDP and PFSA across both basic and advanced airway procedures in undergraduate medical training settings. In fact, to date, no published research has simultaneously evaluated these two methods in the context of both basic airway techniques, such as LMA placement and ETI, and complex procedures like fiberoptic bronchoscope-guided intubation.

This study aims to compare the impact of RCDP and PFSA on skill acquisition and performance in three key airway management procedures, with the objective of identifying the most effective methodology for novice learners in a massed practice format, while accounting for procedural complexity. The findings may help guide academic institutions in selecting time-efficient, evidence-based training strategies that can be applied across various clinical learning environments.

## Materials and methods

Participants

A total of 60 third-year medical students with no prior theoretical or practical experience in airway management procedures were enrolled in the study. One participant was excluded due to absenteeism, resulting in 59 participants included in the final analysis. All participants were enrolled in the Clinical Simulation and Prehospital Trauma module at the Universidad de Especialidades Espíritu Santo.

Training program

The study was carried out in two separate instructional cohorts, each consisting of 30 medical students. The first cohort, trained between October and November 2024, received simulation-based instruction using the RCDP method. The second cohort, trained between April and May 2025, followed PFSA. The inclusion of the Peyton-trained cohort aimed to provide a comparative framework against RCDP, using a well-established teaching strategy in medical education. Peyton’s method is known to support improved learning outcomes, especially in small-group formats with low instructor-to-student ratios [9]. In both cohorts, instruction was delivered by an expert intensivist (K.H.B.C) and a physician trained in airway management (K.H.B.Z) to ensure consistency in content delivery across both methodologies. The training consisted of three sessions, each lasting two and a half hours. During these sessions, the instructor taught three airway management techniques. Two of them were categorized as basic procedures: LMA placement and ETI. The third, FOI, was considered an advanced technique. For each procedure, the training covered technical execution, clinical indications, preparation steps, equipment usage, induction sequence, and verification of correct placement. The training was conducted over a three-week period, with each week dedicated to one specific airway management technique. To accommodate all participants, each week was divided into six small-group sessions of five students each. This structure ensured that every student received individualized, hands-on instruction. The time allocated per participant depended on the complexity of the procedure. In Week 1, students practiced LMA placement with 20 minutes of individual training. In Week 2, ETI was practiced for 25 minutes per student. In Week 3, students were trained in FOI, also with 25 minutes of practice per student.

Evaluation scenario

**Figure 1:**
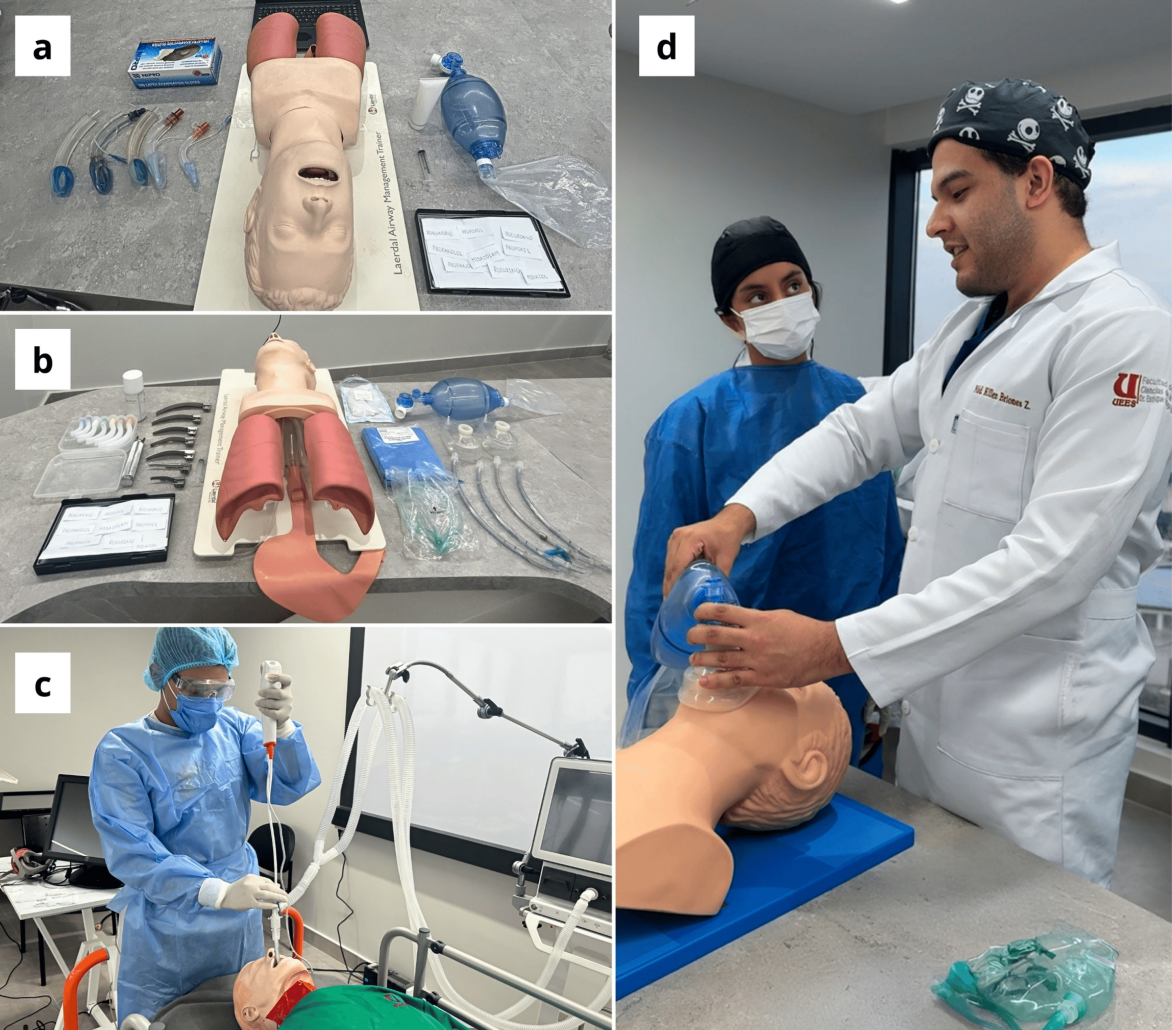
Simulation scenarios and educational setup (a) Laryngeal mask airway (LMA) scenario. The setup includes the Laerdal^®^ Airway Management Trainer, a selection of laryngeal masks, a bag-valve mask, and pharmacological cards used to assess medication selection and dosage accuracy during simulation. (b) Endotracheal intubation (ETI) scenario. The equipment includes multiple laryngoscope blades, endotracheal tubes, airway adjuncts, and printed drug options, including intentionally incorrect spellings, to evaluate the participant’s ability to recognize and calculate proper induction medications. (c) Fiberoptic intubation (FOI) scenario. A participant performs FOI using the Ambu^®^ aScope 4 Broncho and a SimMan^®^ 3G manikin under simulated difficult airway conditions. (d) Endotracheal intubation (ETI) training. A student explains and demonstrates the preoxygenation step to the instructor using a bag-valve mask and airway trainer, as part of Step 3 of Peyton’s Four-Step Approach, where the learner teaches back the procedure under guided supervision.

The final evaluation was conducted in Week 4 for all participants. Assessments were carried out by an intensivist (J.B.S.) who was blinded to group allocation and had not been involved in the training phase. The first and second stations assessed LMA placement and ETI using the Laerdal Airway Management Trainer (Laerdal Medical AS, Stavanger, Norway) (Figures 1a, 1b). In both stations, students were asked to select the appropriate size of the endotracheal tube, the laryngoscope blade, and the LMA according to the patient’s height and weight. They were also required to identify the correct pharmacological agents for induction, with several options intentionally containing incorrect or misspelled names. Students were instructed to select the correct medication and to calculate the appropriate drug dosage. The third station assessed FOI using an Ambu^®^ aScope 4 Broncho (Ambu, Copenhagen, Denmark) and a SimMan^®^ 3G manikin (Laerdal Medical AS) (Figure 1c). Students were given a maximum of nine minutes to complete each procedure.

Three clinical simulation scenarios were presented during the evaluation. The first involved a 38-year-old female radio presenter scheduled for a left hallux valgus correction. Her American Society of Anesthesiologists (ASA) physical status classification was II, and she weighed 60 kg. She reported smoking approximately five cigarettes per day for the past 12 years, had no known drug allergies, and had previously undergone a right foot arthroplasty under general anesthesia without complications. For this procedure, she opted for airway management using an LMA. The second case described a 36-year-old male physical education teacher scheduled for a minimally invasive repair of a right Achilles tendon rupture. His ASA classification was II, and he weighed 84 kg. He regularly used oral nicotine pouches for the past eight years, had no history of surgery, and reported no known allergies. General anesthesia with ETI was planned. The final case focused on a 52-year-old male accountant scheduled for an elective laparoscopic cholecystectomy due to symptomatic gallstones. He had a body mass index (BMI) of 47.3 (weight: 132 kg, height: 1.67 m), consistent with morbid obesity. His medical history included obstructive sleep apnea diagnosed three years earlier, currently managed with nocturnal continuous positive airway pressure (CPAP) therapy. He had no known drug allergies or prior anesthesia-related complications. His ASA physical status classification was III. Due to the anticipated difficulty in airway management, FOI under general anesthesia was planned.

Assessment tool

An Objective Structured Assessment of Technical Skills (OSATS) tool was used to evaluate both LMA placement and ETI. The checklist covered key aspects of the procedure, including preparation, rapid induction, device placement, and verification [10]. For LMA placement, the checklist included 11 steps, each rated on a Likert scale from 1 to 5, resulting in a minimum score of 11 and a maximum score of 55. For reliability analysis, the scale was divided into two dimensions: verification and insertion technique. For ETI, the checklist consisted of 18 steps, also rated on a 1-to-5 Likert scale, with total scores ranging from 18 to 90. This scale was divided into three dimensions for reliability analysis: execution, preparation, and verification. For FOI, a Global Rating Scale (GRS) was used to assess bronchoscope handling and movement quality. In addition, a specific checklist with 10 dichotomous items (performed correctly or incorrectly) was used to evaluate the intubation procedure itself, with a minimum score of 0 and a maximum of 10 points [11]. To classify participants’ performance, three levels of competence were established: non-competent, competent, and mastery. The cutoff thresholds for these categories were defined a priori based on percentage ranges of the total score for each checklist: <60% (non-competent), 60%-84% (competent), and ≥85% (mastery). These thresholds were subsequently reviewed and validated by a panel of three expert intensivists using a modified Angoff method. Each expert independently assessed whether the proposed ranges accurately reflected the minimum expected performance of novice, competent, and mastery-level learners. For LMA placement (maximum score = 55), the validated thresholds were <33 points (non-competent), 33-46 points (competent), and ≥47 points (mastery). For ETI (maximum score = 90), performance was classified as <54 points (non-competent), 54-76 points (competent), and ≥77 points (mastery). For FOI (maximum score = 10), thresholds were defined as <6 points (non-competent), 6-8 points (competent), and ≥9 points (mastery). These tiered classifications allowed for a more granular assessment of technical skill acquisition across procedures of varying complexity, while ensuring that scoring standards were informed by expert clinical judgment.

Ethics

This study was exempted from full ethical review by the Ethics Committee of the Universidad de Guayaquil (UG-CEISH-2024-0060), as it involved no personal or sensitive data and focused solely on educational simulation outcomes. Informed consent was obtained from all participants. The project forms part of a broader initiative within the Medical Education in Simulation Program at Universidad de Especialidades Espíritu Santo, which promotes innovative, low-risk educational research.

Statistical analysis

The Shapiro-Wilk test was used to assess the normality of data distribution between groups. When the data were normally distributed, independent sample t-tests were performed to compare total OSATS scores. For non-normally distributed data, the Mann-Whitney U test was used. To analyze proficiency levels between groups, the Chi-squared test or Fisher’s exact test was applied, depending on expected cell frequencies. Cronbach’s alpha was used to evaluate the internal consistency of the assessment tools in this sample, and item-total correlations were calculated to examine the grouping of dimensions. A p-value < 0.05 was considered statistically significant. All statistical analyses were performed using MedCalc® Statistical Software version 23.1.7 (MedCalc Software Ltd, Ostend, Belgium; https://www.medcalc.org; 2025).

## Results

A total of 60 medical students completed the study, with a demographic distribution of 70% female (n = 42) and 30% male (n = 18). The mean age of participants was 20.3 ± 1.96 years.

Laryngeal mask airway

**Figure 2:**
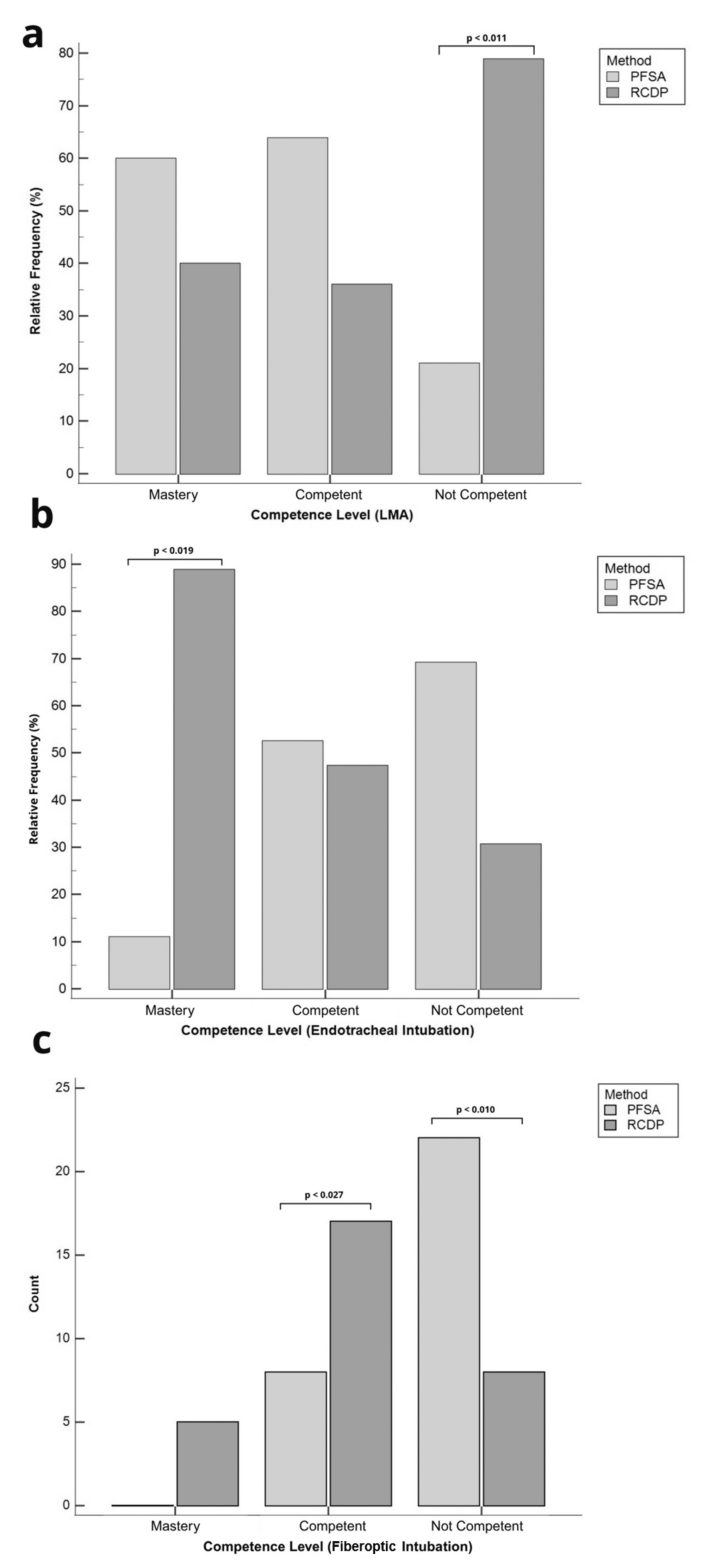
Comparison of competence levels by training method across airway procedures Panels (a) and (b) display the distribution of participants by performance level as relative frequency (%), while panel (c) displays the absolute count (N). Statistical comparisons were performed using the Chi-squared test (χ²), with the corresponding test statistic and degrees of freedom (df) reported in the main text. Statistical significance was defined as p < 0.05. In panel (a), corresponding to the laryngeal mask airway (LMA), a significantly higher proportion of not competent participants was observed in the RCDP group (p = 0.009). In panel (b), representing endotracheal intubation (ETI), a significantly greater proportion of mastery-level participants was observed in the RCDP group (p = 0.013). In panel (c), related to fiberoptic intubation (FOI), significant differences were found across all three performance levels, including a greater proportion of competent participants and a lower proportion of not competent participants in the RCDP group (p = 0.027 and p = 0.010, respectively). PFSA: Peyton’s Four-Step Approach; RCDP: Rapid Cycle Deliberate Practice

For laryngeal mask placement training, the group trained using the PFSA method demonstrated significantly higher technical performance on the OSATS (38.06 ± 6.67 vs. 31.93 ± 7.70; t = -3.30, df = 58, p = 0.0017) (Table 1). However, no significant differences were found regarding the proportion of participants who achieved mastery level in either group (PFSA: 3/30 (10.0%) vs. RCDP: 2/30 (6.7%)). A significant difference was observed in the distribution of competence levels between groups (χ² = 9.35, df = 2, p = 0.0093). Specifically, a higher proportion of participants in the RCDP group were classified as not competent (15/30 (50.0%)) compared to the PFSA group (4/30 (13.3%); χ² = 6.36, df = 1, p = 0.011) (Figure 2a). The internal consistency of the assessment tool was high, with Cronbach’s alpha (α) of 0.70.

**Table 1:** Comparison of performance scores between PFSA and RCDP in LMA placement, ETI, and FOI, using OSATS, GRS, and procedural checklists Data are presented as mean ± SD for normally distributed variables and median (IQR) for non-normally distributed variables. Statistical comparisons were performed using independent-sample t-tests (with degrees of freedom (df)) for parametric variables and the Mann–Whitney U test (with standardized Z values) for non-parametric data. The corresponding test statistic (t or U) is shown alongside each comparison. Statistical significance was set at p < 0.05. GRS: Global Rating Scale; IQR: interquartile range; SD: standard deviation; OSATS: Objective Structured Assessment of Technical Skills; PFSA: Peyton’s Four-Step Approach; RCDP: Rapid Cycle Deliberate Practice

Procedure	Assessment tool	Method	N	Score	Test statistic	p-value
Laryngeal mask airway (LMA)	OSATS (mean ± SD)	PFSA	30	38.06 ± 6.67	t = –3.30, df = 58	0.0017
		RCDP	30	31.93 ± 7.70		
Endotracheal intubation (ETI)	OSATS (mean ± SD)	PFSA	30	58.90 ± 11.87	t = 2.59, df = 58	0.012
		RCDP	30	67.00 ± 12.32		
Bronchoscope handling	GRS (mean ± SD)	PFSA	30	9.66 ± 2.79	t = 5.11, df = 58	<0.0001
		RCDP	30	14.73 ± 4.65		
Fiberoptic intubation (FOI)	Checklist (median (IQR))	PFSA	30	4.0 (3–6)	U = 244.0, Z = –3.08	0.0021
		RCDP	30	6.0 (5–7)		

Endotracheal intubation

For ETI, the RCDP group achieved significantly higher OSATS scores compared to the PFSA group (67.00 ± 12.32 vs. 58.93 ± 11.87; t = 2.59, df = 58, p = 0.012) (Table 1). A significant difference was also observed in the overall distribution of proficiency levels between groups (χ² = 7.47, df = 2, p = 0.0238). Specifically, a greater proportion of participants in the RCDP group reached the mastery level (8/30 (26.7%)) compared to the PFSA group (1/30 (3.3%); χ² = 5.44, df = 1, p = 0.019). No statistically significant differences were found in the proportion of competent participants (PFSA: 20/30 (66.7%) vs. RCDP: 18/30 (60.0%); χ² = 0.10, df = 1, p = 0.745) or non-competent participants (PFSA: 9/30 (30.0%) vs. RCDP: 4/30 (13.3%); χ² = 1.92, df = 1, p = 0.165) (Figure 2b). The internal consistency of the assessment instrument was high (α = 0.86). The item-total correlations for the variables execution, preparation, and verification were 0.8110, 0.7616, and 0.6963, respectively.

Bronchoscope handling and FOI

For bronchoscope handling, the RCDP-trained group demonstrated significantly higher GRS scores compared to the PFSA group (14.73 ± 4.65 vs. 9.66 ± 2.79; t = 5.11, df = 58, p < 0.0001). Similarly, for FOI, the RCDP group achieved higher scores on the procedural checklist than the PFSA group (6.0 (5-7) vs. 4.0 (3-6); U = 244.0, Z = -3.08, p = 0.0021) (Table 1). A significant difference was observed in the distribution of competence levels between groups (χ²(2) = 14.77, p = 0.0006). Although a higher proportion of participants in the RCDP group reached the mastery level (5/30 (16.7%)) compared to the PFSA group (0/30 (0%)), this difference did not reach statistical significance (p = 0.052). However, the proportion of competent participants was significantly greater in the RCDP group (17/30 (60.0%)) than in the PFSA group (8/30 (23.3%); χ²(1) = 3.24, p = 0.027). Additionally, a significantly lower proportion of non-competent participants was observed in the RCDP group (8/30 (26.7%)) compared to the PFSA group (22/30 (73.3%); χ²(1) = 6.53, p = 0.010) (Figure 2c). The internal consistency was high, with α = 0.90 for the GRS and α = 0.77 for the FOI checklist.

## Discussion

Our findings reveal that students trained with PFSA demonstrated significantly higher technical proficiency, as measured by OSATS scores, compared to those trained with RCDP. Although no significant differences were found in the proportion of participants who achieved overall proficiency, a significantly higher number of non-competent participants was observed in the RCDP group. The observed superiority of PFSA in LMA placement training may be attributed to its structured, stepwise approach emphasizing demonstration, deconstruction, comprehension, and performance as well as its alignment with spaced learning principles, which incorporate intentional intervals and repeated practice to enhance retention and mastery and have been proven to statistically improve knowledge retention among medical students [12]. This methodology allows learners to develop a comprehensive understanding of each component of the procedure before attempting it in its entirety, which has been shown to enhance both cognitive and psychomotor learning in medical education [13]. Our results are consistent with previous studies that have highlighted the effectiveness of PFSA particularly among novice learners [14].

Conversely, in the context of ETI training, medical students trained using RCDP achieved significantly higher OSATS scores, reflecting improved procedural choreography and technical proficiency. Regarding proficiency levels, a significantly greater proportion of participants in the RCDP group reached the mastery level compared to the PFSA group, further supporting the effectiveness of this approach in developing high-level performance. Our results concur with different studies where RCDP positively contributed to the teaching and skill acquisition of ETI. For example, Gross et al. found that participants receiving RCDP training showed a significantly greater improvement in intubation checklist scores compared to those who received feedback only after the simulation, indicating enhanced skill acquisition during the procedure [15]. These findings suggest that the iterative nature of RCDP, characterized by immediate feedback and repeated simulation-based practice, facilitates the prompt correction of errors and the reinforcement of correct techniques, thereby optimizing learning in more complex and sequential procedures such as ETI.

In our study, medical students trained with RCDP demonstrated better performance in FOI compared to those trained with the PFSA method, including improved handling of the bronchoscope. However, none of the participants achieved the mastery level in this procedure. This outcome may be attributed to the inherent complexity of FOI, which requires advanced visuospatial skills, refined hand-eye coordination, and a longer duration of training to achieve proficiency [16,17]. Nevertheless, the proportion of competent participants was significantly higher in the RCDP group compared to the PFSA group (p = 0.0194), and the proportion of non-competent participants was significantly lower in the RCDP group (p = 0.0003). These findings suggest that RCDP may offer advantages in accelerating skill development even in high-complexity procedures such as FOI. In this context, a recent study by Wu investigated the effectiveness of a multimodal approach that combined the flipped classroom model with PFSA to train anesthesia residents in orotracheal intubation using a bronchoscope. Their results demonstrated that this structured strategy outperformed traditional methods, leading to higher satisfaction levels and improved outcomes in both theoretical and practical assessments. Peyton’s method facilitated step-by-step internalization of the technique, helping residents progress from observation to independent performance with increased confidence and precision [18].

Similarly, Wolf et al. reported that the use of PFSA significantly enhanced performance and success rates in fiberoptic-assisted tracheoscopy using a neonatal simulator, particularly among participants without prior endoscopic experience. However, in contrast to these findings, our study showed that RCDP was more effective than PFSA in medical students. This discrepancy may be explained by differences in the learners' clinical backgrounds, the procedural complexity, or the nature of the simulation environments. These contrasts underscore the importance of aligning instructional strategies with both the experience level of learners and the technical demands of the procedure being taught [19].

Our findings suggest that RCDP may offer a promising alternative by combining immediate feedback with repetitive hands-on practice, potentially addressing the skill retention challenges posed by this low-frequency, high-complexity procedure. There is currently no available information evaluating the use of RCDP for training in FOI. Despite RCDP’s well-documented effectiveness in other airway management procedures such as orotracheal intubation and laryngeal mask placement, its application to fiberoptic techniques has remained largely unexplored.

These findings highlight the value of tailoring simulation-based training methods to the specific demands of clinical procedures. While PFSM proved more effective in enhancing technical proficiency in LMA placement, likely due to its structured, stepwise pedagogy and alignment with spaced learning principles, RCDP yielded superior outcomes in ETI training and FOI, underscoring the benefits of its iterative, feedback-driven format. These findings support the strategic use of differentiated instructional approaches in medical education, suggesting that the choice of training modality should be informed not only by the cognitive and procedural complexity of the skill being taught but also by learners’ experiences and preferences, as identified through targeted surveys or interviews.

From an institutional perspective, both PFSA and RCDP were implemented in a massed practice format, enabling participants to reach at least the competent threshold in LMA placement and ETI within a condensed training schedule. This intensive delivery model requires fewer total sessions and less prolonged faculty engagement compared with distributed practice programs, which, although potentially advantageous for long-term retention, demand greater time and resource investment. In time-constrained curricula or resource-limited settings, the balance of educational benefit against operational cost may favor massed practice for achieving short-term competency in procedures such as LMA placement and ETI. However, for complex skills like bronchoscope handling in FOI, where mastery requires advanced visuospatial coordination and refined psychomotor control, a distributed practice format could be more appropriate to support progressive skill consolidation and long-term retention.

Limitations

This study has some limitations. First, it was conducted in a single academic institution, which may limit the generalizability of the findings to other educational settings. Second, the training was delivered in a massed practice format, which, while aligned with the study’s objective of evaluating instructional effectiveness in a time-constrained setting, does not allow for conclusions regarding long-term skill retention. Although the aim was to assess short-term acquisition under intensive conditions, future studies could explore the durability of learning over time to better understand retention. Additionally, randomization of participants was not feasible due to the fixed scheduling and periodicity of the academic course. A randomized design would have strengthened the internal validity of the study and should be considered in future research.

## Conclusions

This simulation-based study compared two instructional methodologies: PFSA and RCDP in the training of undergraduate medical students across basic and advanced airway management procedures. The results indicate that PFSA was more effective for LMA placement, likely due to its structured, stepwise framework and alignment with spaced learning principles. In contrast, RCDP demonstrated superior outcomes in ETI and FOI, suggesting its advantage in teaching more complex and sequential procedures through immediate feedback and repeated hands-on practice. These findings support the integration of tailored educational strategies based on the complexity of the task and the experience level of learners. By aligning instructional methods with specific procedural demands, medical educators can enhance the efficiency and effectiveness of simulation-based airway training, particularly in time-constrained academic environments.
